# The impact of statins on health services utilization and mortality in older adults discharged from hospital with ischemic heart disease: a cohort study

**DOI:** 10.1186/1472-6963-9-198

**Published:** 2009-11-04

**Authors:** Charmaine A Cooke, Susan A Kirkland, Ingrid S Sketris, Jafna Cox

**Affiliations:** 1 Population Health Research Unit, Dalhousie University, 5790 University Avenue, Halifax, Nova Scotia, B3H 1V7, Canada; 2 Department of Community Health and Epidemiology, Faculty of Medicine, Dalhousie University, 5849 University Avenue, Halifax, Nova Scotia, B3H 4H7, Canada; 3 College of Pharmacy, Dalhousie University, 5968 College Street, Halifax, Nova Scotia, B3H 3J5, Canada; 4 Division of Cardiology, Capital District Health Authority, 2ndFloor, Halifax Infirmary Hospital, 1796 Summer Street, Halifax, Nova Scotia, B3H 3A7, Canada

## Abstract

**Background:**

Cardiovascular disease (CVD) carries a high burden of morbidity and mortality and is associated with significant utilization of health care resources, especially in the elderly. Numerous randomized trials have established the efficacy of cholesterol reduction with statin medications in decreasing mortality in high-risk populations. However, it is not known what the effect of the utilization of these medications in complex older adults has had on mortality and on the utilization of health services, such as physician visits, hospitalizations or cardiovascular procedures.

**Methods:**

This project linked clinical and hospital data from the Improving Cardiovascular Outcomes in Nova Scotia (ICONS) database with administrative data from the Population Health Research Unit to identify all older adults hospitalized with ischemic heart disease between October 15, 1997 and March 31, 2001. All patients were followed for at least one year or until death. Multiple regression techniques, including Cox proportional hazards models and generalized linear models were employed to compare health services utilization and mortality for statin users and non-statin users.

**Results:**

Of 4232 older adults discharged alive from the hospital, 1629 (38%) received a statin after discharge. In multivariate models after adjustment for demographic and clinical characteristics, and propensity score, statins were associated with a 26% reduction in all- cause mortality (hazard ratio (HR) 0.74, 95% confidence interval (CI) 0.63-0.88). However, statin use was not associated with subsequent reductions in health service utilization, including re-hospitalizations (HR, 0.98, 95% CI 0.91-1.06), physician visits (relative risk (RR) 0.97, 95% CI 0.92-1.02) or coronary revascularization procedures (HR 1.15, 95% CI 0.97-1.36).

**Conclusion:**

As the utilization of statins continues to grow, their impact on the health care system will continue to be important. Future studies are needed to continue to ensure that those who would realize significant benefit from the medication receive it.

## Background

Although mortality from cardiovascular disease (CVD) has been decreasing steadily in Canada, it is still the leading cause of death, accounting for 33% of all mortality in 2003 [1-3]. Patients with CVD have a high burden of morbidity and utilize significant health care resources, resulting in high health care costs. In 1998, CVD represented the most costly diagnostic category, accounting for a total cost of $18.5 billion in Canada (11.6% of total costs of all illnesses) [3,4].

In several randomized controlled trials (RCTs) [5-12], the use of statins has been shown to substantially reduce the occurrence of morbidity and mortality in people with coronary artery disease. Consistent treatment effects across multiple groups, including older adults [13-17] have been demonstrated. The evidence on the efficacy of statins from these RCTs has led to widespread use of these medications in all age groups [18-20].

Gaps in evidence may exist on the safety and effectiveness of drugs during real world use where drugs may be often used by diverse patient groups outside the controlled environment of clinical trials [21-23]. In particular, older adults who may have many concomitant diseases, complex drug regimens and cognitive and functional decline are often excluded from RCTs; this may limit the generalizability of RCTs to these populations. Therefore, the impact of statins on mortality and health service utilization in the real world in older adults may need to be explored.

The purpose of this study was to determine if older adults who received statins after discharge from hospital would have better health outcomes (less use of health services and decreased mortality) compared to those who did not receive statins.

## Methods

### Data Sources

The Improving Cardiovascular Outcomes in Nova Scotians (ICONS) project was a multi-stakeholder, province-wide initiative that first enrolled patients on October 15, 1997 [24]. It includes a registry of all patients in Nova Scotia, Canada hospitalized with a diagnosis of ischemic heart disease (IHD), congestive heart failure or atrial fibrillation [25]. Data for the ICONS study was retrospectively abstracted from patient charts; variables include patient demographics, laboratory and diagnostic tests, social history, cardiovascular risk factors, medical history, in-hospital cardiovascular procedures and admission and discharge medications.

The Population Health Research Unit (PHRU), Dalhousie University, houses population-level administrative health data for the province of Nova Scotia [26]. The data repository contains comprehensive information about insured health services delivered to residents of Nova Scotia from 1989 onward. The databases at PHRU contain information on vital statistics, physician billings, hospitalizations and all prescriptions dispensed to eligible adults 65 years and over registered in the Nova Scotia Pharmacare Program.

### Study Cohort and Design

A retrospective cohort design was used. The study population included all individuals ≥ 66 years old registered in the Nova Scotia Seniors Pharmacare Program (NSSPP) who were discharged alive from hospital in Nova Scotia, Canada between October 15, 1997 and March 31, 2001, with a diagnosis of IHD. The NSSPP is a publicly funded drug insurance plan that reimburses drugs and medical supplies listed in the Nova Scotia Formulary for entitled seniors in the province. The beneficiaries of this program are seniors with a minimum age of 65 years who enrolled in the program by paying the required insurance premium and the co-payments. The NSSPP does not provide coverage for senior residents who have private drug insurance, benefit coverage with Veterans Affairs Canada or First Nations and Inuit Health. Approximately 85% of seniors in Nova Scotia are eligible beneficiaries under the NSSPP; the remainder of the senior population has drug coverage under private or Federal programs (Data on file, Population Health Research Unit, Dalhousie University). Patients were followed for at least one year or until death; if a patient had more than one hospital admission during the study period, the first hospitalization date was used as the index hospitalization. This study received approval from the Research Ethics Board of the Capital Health District Health Authority, Halifax, Nova Scotia.

### Measures of Outcomes and Exposures

The primary outcome of interest was defined as the utilization of health services (physician services, hospitalizations, or a coronary revascularization procedure) after the index discharge from hospital with IHD, and within the follow-up period (to March 31, 2002 or death). The secondary outcome measure was all-cause mortality after the index hospitalization.

The primary exposure of interest was the dispensing of at least one prescription for a statin medication after discharge from hospital as identified through the Nova Scotia Pharmacare databases. Non-initiators of statin medications were defined as those patients receiving no statin prescriptions for at least one year before hospital admission and throughout the study period. New initiators of statin medications were defined as those patients who received at least one prescription for a statin after hospital discharge. Statins were identified using Drug Identification Numbers (DINs), a Canadian system used to identify unique drug products and through the Anatomical Therapeutic Chemical (ATC) classification system [27,28].

### Statistical Analysis

#### Descriptive Analysis

Descriptive statistics were used to compare baseline patient characteristics between groups using chi-squared tests for categorical variables and Student t-tests for continuous variables. Bivariate associations were examined with Mantel Haenszel summary odds ratios to determine possible interactions or effect modification between statin therapy, mortality and age or gender. No age or gender interactions were identified.

The number of distinct medications prescribed in the previous year (NODMP) is one method used to adjust for co-morbidity in administrative database studies [29-36]. As the NODMP is easy to access and calculate and has been validated [33,35,37-39], and as health service utilization was the primary outcome measure for this project, the NODMP was used as the method to adjust for co-morbidity.

#### Propensity Model

In observational studies, there is no control over treatment and the exposed and unexposed groups may have large differences in their observed covariates, which lead to biased estimates of treatment effects [40,41]. Propensity analysis attempts to identify patients who are similar except for their treatment assignment to approximate the conditions of a randomized controlled trial [40-44].

A multivariate logistic regression model was generated to estimate for each patient the propensity to receive a statin after discharge from hospital. Available clinical, demographic and treatment variables were incorporated into the model. The variables included in the propensity score model are listed in Table 1. The ability of the model to discriminate between patients who did and did not receive statin therapy was assessed with the c-statistic. The study population was divided into quintiles of increasing probability for statin initiation, and the propensity score was entered by quintile level into the final multivariate regression models used to determine the relationship between statins and the outcomes of interest [41-44].

**Table 1:** List of Variables entered into the Propensity Model and their Description

Variable	Measure	Source
**Outcomes and Exposures**		
Physician Visits	Continuous	PHRU physician services database
Hospitalizations	Continuous	PHRU hospitalization database
Coronary Re-vascularizaton	Continuous	ICONS (Inpatient chart)
Mortality	Yes/No	ICONS (Vital Statistics)
**Demographics**		
Age	Continuous Categorical: 66-75, 75-80, >80 (by tertiles)	PHRU (Pharmacare database)
Gender	Male, Female	ICONS (Inpatient chart)
Region of Province where Patient Resides	Northern, Eastern, Western, Southern	ICONS (Inpatient chart)
*Risk Factors*		
Total blood cholesterol measured in hospital	Yes/No	ICONS (Inpatient chart)
LDL cholesterol measured in hospital	Yes/No	ICONS (Inpatient chart)
HDL cholesterol measured in hospital	Yes/No	ICONS (Inpatient chart)
Triglycerides measured in hospital	Yes/No	ICONS (Inpatient chart)
Diabetes	Yes/No	ICONS (Patient reported)
Hypertension	Yes/No	ICONS (Patient reported)
Smoking	Past, Current, Never	ICONS (Patient reported)
Family History of CVD	Yes/No	ICONS (Patient reported)
Previous PTCA	Yes/No	ICONS (Patient reported)
Previous CABG	Yes/No	ICONS (Patient reported)
Previous CHF	Yes/No	ICONS (Patient reported)
Previous Cardiac Catheterization	Yes/No	ICONS (Patient reported)
Previous Myocardial Infarction	Yes/No	ICONS (Patient reported)
Previous stroke	Yes/No	ICONS (Patient reported)
Previous TIA	Yes/No	ICONS (Patient reported)
Year of Entry into study	By Quartiles	ICONS (Inpatient chart)
Number of distinct prescription medications in 6 months pre-hospitalization	Continuous Categorical: <3, 3-<6, 6-<9, ∃ 9 (by quartiles)	PHRU Pharmacare database
**In-hospital clinical data**		
Discharge diagnosis	UA/AMI	ICONS (Health Records)
In-hospital procedures (PTCA, CABG, EST, Cardiac catheterization)	Yes/No	ICONS (Inpatient Chart)
Medications on discharge (ASA, Beta-blockers, CCB, ACE, Fibrates)	Yes/No	ICONS (Inpatient Chart)

#### Multivariate Analysis

Once propensity scores had been estimated for each patient, the scores were sorted in ascending order, and then stratified into quintiles. Propensity scores by quintiles were fairly well matched between statin initiators and non-initiators. As the goal of stratification is to remove differences in covariates between the two groups, covariates were examined within each quintile for statin initiators and non-initiators to ensure that the two groups were well matched for each covariate; any variables that were not balanced within a quintile were included in the final multivariate models as first order interaction terms. Other variables forced into the final multivariate models included age and gender.

We determined the impact of the number and rate of re-hospitalizations using generalized linear models (GLM) and Cox proportional hazards models. GLM was also used to examine the relationship between statins and the number of physician services per person. The negative binomial distribution was used in the GLM models as the Poisson assumption was violated. Cox proportional hazards models were used to estimate the HR and 95% CI for the association between coronary revascularizations statins and mortality and statins. All multivariate analyses were undertaken both with and without propensity adjustment.

All statistical analysis was undertaken using the SAS statistical computer program version 8.2 [45] and models were developed using a non-automated, backwards selection process.

## Results

### Baseline Characteristics

The study population consisted of 4232 older adults discharged alive from the hospital with a diagnosis of IHD between October 15, 1997 and March 31, 2001. The mean age was 77.5 years (± 7 years), and 48% (n = 2036) were male. A total of 38% of patients (n = 1629) were dispensed a statin during the study period. Overall, patients were followed for an average of 28.0 (± 14.6 months) months from discharge, during which time 26% of patients (n = 1120) died. The percentage of those receiving statins remained relatively stable over the study period, with annual rates of utilization of 39.1%, 38.8%, 37.7% and 38.4% (p = 0.92). Over 50% (52.7%) of patients received their first statin prescription within 30 days of hospital discharge, 19.5% of patients received their first prescription for a statin within 90 days of discharge, 17.4% within 365 days, and 10.4% received the first statin prescription 365 days after discharge.

The demographic and clinical characteristics of the 4232 patients in the cohort are described in Table 2. There were notable differences between statin initiators and non-initiators. On index hospital admission, statin initiators seemed healthier in that they were younger, less often had a history of a CVD, and had fewer prescription medications dispensed in the six months pre-hospitalization. However, they were more likely to be current smokers and to have a family history for CVD. During the index hospitalization, statin initiators more often received a coronary revascularization procedure, and were more often prescribed cardiovascular medications on discharge. The c-statistic for the propensity model was 0.78, indicating that the propensity model discriminated acceptably between patients receiving a statin on discharge and those who did not [44]. A model with a c-statistic of 0.5 would reflect a completely random prediction model, while a model that discriminates perfectly between patients with and without an event would have a c-statistic of 1.0.

**Table 2:** Characteristics of 4232 Nova Scotia seniors discharged from hospital with unstable angina or myocardial infarction

Characteristic	Statin Dispensed (n = 1629)	No Statin Dispensed (n = 2603)	p value
**Demographics**			
Age (years) (range)	74.6 ± 5.7 (66-98 years)	79.2 ± 7.1 (66-101 years)	< 0.0001
Gender (Males)	49.8%	47.1%	0.085
*Risk Factors/Comorbidities*			
Hypertension	58.8%	58.2%	0.705
Smoking			< 0.0001
Never	48.9%	57.9%	
Current	18.00%	13.5%	
Past	33.1%	28.6%	
Family history of CVD	15.3%	8.4%	< 0.0001
Diabetes	26.2%	29.0%	0.047
History of CHF	8.5%	17.6%	< 0.0001
History of MI	25.8%	30.9%	0.0004
History of Stroke	6.4%	11.5%	< 0.0001
History of TIA	4.5%	7.5%	< 0.0001
Number of distinct prescription medications in 6 months pre-hospitalization	5.7 ± 4.2	6.6 ± 4.6	< 0.0001
*Area in Province Patient Resides*			0.035
North	18.1%	17.6%	
Central	35.1%	32.5%	
East	21.7%	20.8%	
West	25.0%	29.0%	
**Factors During Index Hospitalization**			
*Discharge Diagnosis*			
Unstable Angina	46.0%	51.0%	0.001
Myocardial Infarction	54.0 %	49.0%	
*In-hospital procedures*			
CABG	6.0%	3.7%	0.0004
Cardiac catheterization	27.1%	15.6%	< 0.0001
PTCA	10.8%	5.7%	< 0.0001
EST	48.8%	25.1%	< 0.0001
*Concomitant medications written at hospital discharge*			
ASA	85.2%	75.3%	< 0.0001
ACE inhibitor	46.2%	47.6%	0.375
Beta-blocker	83.1%	75.9%	< 0.0001
Calcium channel blocker	37.4%	37.5%	0.950
Fibrates	6.1%	4.9%	0.0092
*Cholesterol Levels measured in Hospital (Y/N)*			
Total cholesterol	68.1%	46.0%	< 0.0001
LDL cholesterol	63.1%	42.0%	< 0.0001
HDL cholesterol	50.9%	49.1%	< 0.0001
Triglycerides	54.5%	45.5%	< 0.0001
**Factors Post-Index Hospitalization**			
*Follow-up (# years)*	2.7 ± 1.1	2.1 ± 1.2	< 0.0001
*Crude Mortality*	11.4%	35.9%	< 0.0001

### Impact of Statins on Health Services Utilization

Although the percentage of older adults re-admitted to the hospital for any cause was decreased by 16% (HR 0.84, 95% CI 0.78-0.90) before propensity adjustment, this beneficial effect disappeared once propensity scores were incorporated into the model (Table 3). Adjusting for propensity score resulted in a similar trend for the impact of statins on the percent of those with CVD-related re-hospitalizations. Before propensity adjustment, statins had no impact on CVD-related re-hospitalization rates in older adults; however, after propensity adjustment there was an 11% increase those with CVD-related re-hospitalizations (HR 1.11, 95% CI 1.02-1.22). The number of all-cause re-hospitalizations and CVD-related re-hospitalizations per senior was not affected by statins use either before or after propensity adjustment (Table 3).

**Table 3:** Hazards ratios and relative risks for the association between statins and health service utilization/mortality in 4232 Nova Scotia seniors discharged from hospital with unstable angina or myocardial infarction

Outcome	Impact of Statins on rate of events modeled using Cox Proportional Hazards (HR, 95% CI) (Statin versus No Statin)	Impact of Statins on number of events, modeled using Generalized Linear Models (RR, 95% CI) (Statin versus No Statin)
All-cause re-hospitalizations		
Modeled without propensity score	0.84 (0.78-0.90)	0.97 (0.90-1.04)
Modeled with propensity score	0.98 (0.91-1.06)	1.04 (0.97-1.12)
CVD re-hospitalizations		
Modeled without propensity score	0.98 (0.90-1.07)	0.90 (0.86-1.12)
Modeled with propensity score	1.11 (1.02-1.22)	1.03 (0.92-1.16)
All physician visits	N/A	
Modeled without propensity score		0.84 (0.79-0.88)
Modeled with propensity score		0.97 (0.92-1.02)
CVD-related physician visits	N/A	
Modeled without propensity score		1.18 (1.09-1.29)
Modeled with propensity score		1.34 (1.23-1.46)
Coronary Revascularizations		N/A
Modeled without propensity score	1.55 (1.30-1.84)	
Modeled with propensity score	1.15 (0.97-1.36)	
Mortality		N/A
Modeled without propensity score	0.41 (0.35-0.49)	
Modeled with propensity score	0.74 (0.63-0.88)	

Although statins decreased the number of physician visits in older adults by 16% before propensity adjustment (RR 0.84, 95% CI 0.79-0.88), this beneficial effect was also removed after adjustment for propensity in the model. Statin use was associated with an 18% increase in the number of CVD-related physician visits (95% CI 1.09-1.29) before propensity adjustment and a 34% increase (95% CI 1.23-1.46) after propensity adjustment (Table 3).

Coronary revascularization procedures were significantly increased in those receiving statins before propensity adjustment (HR 1.55, 95% CI 1.30-1.84). However, in contrast to the other outcome measures, including the propensity in the model negated this increase (Table 3).

### Impact of Statins on Mortality

After adjustment for clinical and demographic variables, patients who received statins had a 59% reduction in mortality compared with patients who did not receive statins (HR 0.41, 95% CI 0.35-0.49). After the propensity score was incorporated into the model, statins were associated with a 26% reduction in mortality (HR 0.74, 95% CI 0.63-0.88) (Table 3).

## Discussion

This study found that older adults receiving a statin after discharge from hospital with IHD realized significant mortality benefits; however, reductions in re-hospitalizations, physician visits, or coronary revascularizations were not demonstrated over the follow-up period of approximately two years.

Thirty-eight percent (38%) of older adults discharged from hospital received statins in our study; this may seem inconsistent with current North American guidelines which suggest that most patients at high-risk for CVD disease should receive treatment with these medications regardless of age [46,47]. The low utilization of statins in our population may be a reflection of the study period (1997-2001), with other studies reporting utilization of statins in older adults with established disease over this time period describing similar prescribing rates [48-51].

This study demonstrated that older adults with increased markers for disease severity were less likely to be dispensed statins after discharge than were their healthier counterparts. Other published studies have also shown consistent treatment biases for the use of statins in the older adults at highest risk for developing CVD [37,39,48,49,52,53]. Although some published studies have reported that females with CVD were treated less often than their male counterparts [18,54-57], our results agree with more recent reports that do not demonstrate this treatment bias [48,58-60].

While there is limited data describing health service utilization associated with statin use, we were somewhat surprised that we did not observe a decrease in health service utilization associated with statins similar to that seen in randomized trials [61-63]. Many authors have postulated various mechanisms, including plaque stabilization and improved endothelial function [64-69] that would support the benefits of statin therapy on a variety of health outcomes, including CVD-related events and coronary revascularizations. However, our results are similar to those demonstrated in two other observational studies that examined the impact of statins on re-hospitalizations [38,70], Perhaps more aggressive management of CVD, reflected by prescribing of statins, results in regular ongoing patient follow-up and management, leading to increases in health services utilization.

There may be other reasons why older adults access health services that may have little to do with ill health or with medication utilization. A vast array of health service utilization influences have been identified including societal factors, availability of physicians and hospital beds, and personal factors [71,72]. Many of these factors may have impacted physician visits, re-hospitalizations and coronary revascularization procedures to a greater degree than the use of statin medications in a real-world population.

While statin therapy was associated with increases in CVD-related re-hospitalizations and CVD-related physician visits, these increases may be correlated. Older adults receiving statins may visit their physician for a CVD-related cause more often than those not receiving statins due to ongoing lipid monitoring or to receive a new statin prescription. Due to these increases in physicians' visits, there could be a "detection bias" in that patients that visit their physician may be more likely to be investigated more aggressively for chest pain and be referred to the hospital for recurrent angina.

After adjustment for clinical and demographic variables, statins were associated with a 26% reduction in mortality (HR 0.74, 95% CI 0.63-0.88) after propensity adjustment. The 26% reduction in mortality associated with statin therapy in older adults with CVD in our study is similar to results demonstrated in two large randomized trials [61-63] and in two meta-analyses [73,74] of trials in older adults taking statins.

There have been few observational studies examining the effect of statins on all-cause mortality in older adults with CVD. One recent observational study of 2626 patients 65 years and older with established CVD living in nursing homes [70] demonstrated a 31% reduction in one-year mortality associated with statins. Nonetheless, as older adults admitted to nursing homes often have a high acuity of illness, it is difficult to determine if the benefits demonstrated could be extrapolated to a community dwelling senior population.

Three other studies [75-77] examined the use of statins in patients discharged from hospital with CHD, with stratification by age to determine mortality in older adults. While significant reductions in mortality were demonstrated (28%-60%), these studies did not incorporate propensity analysis techniques into their analysis. One other published study has examined the impact of statins on mortality in older adults discharged from hospital with an AMI that incorporated propensity analysis techniques into the analysis [78]. The authors demonstrated that discharge statin therapy was associated with a decrease in 3-year mortality similar to that found in our study (HR 0.89, 95% CI 0.83-0.96). However, mortality reductions were not evident in those aged 80 years and older. While this age-statin interaction was not demonstrated in our study, this issue needs further exploration.

Several limitations of our study merit consideration. First, statin therapy was not randomly assigned, introducing an important source of bias into the study. Despite adjustments for confounding through multivariate techniques incorporating covariates and propensity adjustment, residual confounding from unobserved or unmeasured confounders cannot be excluded.

Although Canada has a universal health insurance system, access to hospital services, physician services and cardiac procedures has shown to vary with socioeconomic status and across regions [79-81]. While all older adults in this study had coverage for prescription medications through the Nova Scotia Pharmacare Program, prescription co-payments did increase over this study period, from 20% to 33% of each prescription. This may have affected medication access over the study period, although one recent Canadian study disputes this theory [81]. As this study examined older adults discharged from the hospital with IHD, the results may not be generalizable to older adults recruited from primary care. Finally, data was not available on compliance with or discontinuation of statins during the study period. An analysis of these factors may reveal important differences in outcomes depending on the length of time that a person receives the medication. Nonetheless, as our study examines the impact of statin use on health services use and mortality in all statin users, our outcomes reflect those that occur in real-world use of these medications.

## Conclusion

Based on the results of this population-based observational study, we conclude that statins are associated with important reductions in mortality in older adults discharged from hospital with cardiovascular disease. Re-hospitalizations and physician visits were not decreased by statin utilization, however, the number of CVD-related physician visits per senior, and the rate of CVD-related re-hospitalizations were increased, a finding which requires further exploration.

As the utilization of statins continues to grow, their impact on the health care system will continue to be important. Future studies are needed to continue to determine which patients should receive statins, the optimal time from diagnosis to initiation, and strategies to ensure that those who would receive significant benefit from these medications receive it. Further exploration of the relationship between statin use and health services use is required in order to promote safe, appropriate and cost-effective management of older adults with CVD.

## Competing interests

The authors declare that they have no competing interests.

## Authors' contributions

CC, SK, IS and JC made substantial contributions to the design and interpretation of the study. CC performed the statistical analysis. CC and SK drafted the manuscript. All authors read and approved the final manuscript.

## Pre-publication history

The pre-publication history for this paper can be accessed here:

http://www.biomedcentral.com/1472-6963/9/198/prepub
